# Embedding Tree-Based Intrusion Detection System in Smart Thermostats for Enhanced IoT Security

**DOI:** 10.3390/s24227320

**Published:** 2024-11-16

**Authors:** Abbas Javed, Muhammad Naeem Awais, Ayyaz-ul-Haq Qureshi, Muhammad Jawad, Jehangir Arshad, Hadi Larijani

**Affiliations:** 1 Department of Electrical and Computer Engineering, COMSATS University Islamabad, Lahore Campus, Lahore 54000, Pakistan; abbasjaved@cuilahore.edu.pk (A.J.); naeem.awais@cuilahore.edu.pk (M.N.A.); mjawad@cuilahore.edu.pk (M.J.); jehangirarshad@cuilahore.edu.pk (J.A.); 2 Department of Cyber Security and Networks, School of Computing, Engineering and Built Environment, Glasgow Caledonian University, Glasgow G4 0BA, UK; 3 SMART Technology Research Centre, Department of Cyber Security and Networks, School of Computing, Engineering and Built Environment, Glasgow Caledonian University, Glasgow G4 0BA, UK; h.larijani@gcu.ac.uk

**Keywords:** intrusion detection system, embedded ML, TinyML, DoS, MITM, CatBoost

## Abstract

IoT devices with limited resources, and in the absence of gateways, become vulnerable to various attacks, such as denial of service (DoS) and man-in-the-middle (MITM) attacks. Intrusion detection systems (IDS) are designed to detect and respond to these threats in IoT environments. While machine learning-based IDS have typically been deployed at the edge (gateways) or in the cloud, in the absence of gateways, the IDS must be embedded within the sensor nodes themselves. Available datasets mainly contain features extracted from network traffic at the edge (e.g., Raspberry Pi/computer) or cloud servers. We developed a unique dataset, named as Intrusion Detection in the Smart Homes (IDSH) dataset, which is based on features retrievable from microcontroller-based IoT devices. In this work, a Tree-based IDS is embedded into a smart thermostat for real-time intrusion detection. The results demonstrated that the IDS achieved an accuracy of 98.71% for binary classification with an inference time of 276 microseconds, and an accuracy of 97.51% for multi-classification with an inference time of 273 microseconds. Real-time testing showed that the smart thermostat is capable of detecting DoS and MITM attacks without relying on a gateway or cloud.

## 1. Introduction

The Internet of Things (IoT) integrates a wide range of smart objects, devices, and wireless sensor nodes (WSNs) for seamless communication and data exchange. The applications of IoT have been reported in smart homes, smart farming, smart cities, smart wearables, healthcare, education, etc. [1]. It is estimated that the number of IoT devices will surpass 27 billion by 2025 [2]. Billions of IoT devices communicate with each other over the internet and most of the IoT devices have limited memory and computational capabilities, making them vulnerable to a wide range of attacks. These IoT devices are not designed to detect and respond to security attacks [3].

Cybercriminals often target smart homes because they hold a high concentration of valuable digital assets, including sensitive personal information, home security controls, and access to connected devices, such as cameras and locks. These assets are attractive for identity theft, financial fraud, or unauthorized surveillance, making smart homes prime targets [4,5]. These devices are particularly vulnerable to cyberattacks due to occupants’ lack of knowledge, insecure IoT devices, weak security controls, and improper configurations. Based on STRIDE taxonomy, the possible cyberattacks on smart homes are spoofing, tampering, repudiation, information disclosure, DoS, and elevation of privilege [5]. In a DoS attack, the smart device is overwhelmed with traffic by the adversary, and as a result, the device stops working. In an MITM attack, the attacker secretly intercepts the communication between the sender and the receiver without either party being aware [6]. MITM attacks can lead to repudiation and information disclosure attacks.

A smart home is a residence where appliances can be remotely controlled via the internet. The smart home market is projected to grow to USD 633.20 billion by 2032 [7]. This growth is largely driven by the adoption of IoT platforms. Key stakeholders are increasingly focusing on developing smart home appliances that utilize IoT and machine learning. Wifi-based smart thermostats are used in smart homes to control heating and cooling appliances. However, according to Rambus [8], nearly 80% of IoT devices remain exposed to potential cyberattacks, highlighting their significant vulnerability to security breaches. These security threats present major challenges for the expansion of the IoT. Due to security vulnerabilities in IoT devices used in smart homes, hackers can gain unauthorized access to personal information and control of connected appliances. Unauthorized access to the smart thermostat may result in high energy consumption and the occupant’s discomfort.

An IDS for IoT is a system designed to monitor and analyze network traffic to identify and react to intrusions and anomalies, safeguarding the security and integrity of the IoT network. Most of the work on IDS for IoT systems has been simulation-based using existing datasets and real-time implementation has been lacking [9,10,11,12,13,14]. IoT devices have limited computational resources and in many cases, malicious/hacked IoT devices remain undetected and are only detected when they stop working. This could be disastrous for some applications and may result in heavy losses.

Raspberry Pi-based gateways function as intermediary devices that connect local IoT devices to the cloud. They perform essential tasks such as data preprocessing, filtering, and traffic monitoring. Furthermore, these gateways can improve the security of the IoT network by hosting lightweight IDS to detect intrusions and anomalies in real-time. Few authors have implemented IDS on Raspberry Pi-based gateways of smart homes [15,16,17,18] to detect intrusions. However, many commercially available WiFi-based IoT devices of smart homes are connected to the cloud without having Raspberry Pi/computer-based gateways on which IDS can be deployed. Consequently, these IoT devices are easy targets for DoS/Distributed Denial of Service (DDoS) and MITM attacks. Therefore, the security of these IoT devices can be enhanced by embedded ML-based IDS.

Embedded ML, also called TinyML, allows embedding ML models in microcontroller-based IoT devices. Numerous applications of embedded ML/TinyML have recently been reported. A few examples include an intrusion detection system for IoT [19], urban noise data analysis [20], fault diagnosis [21], and soil quality monitoring [22].

Researchers have traditionally implemented IDS on gateways or the cloud due to the availability of network traffic analyzers like Wireshark (https://www.wireshark.org (accessed on 6 October 2024)) and tcpdump (https://www.tcpdump.org/ (accessed on 6 October 2024)). In our recent work [19], we introduced a novel approach by embedding an XGBoost-based IDS for binary classification in an ESP32 (https://www.espressif.com/en/products/socs/esp32 (accessed on 6 October 2024))-powered smart thermostat. The smart thermostat updates the HVAC status, indoor air temperature, and humidity on a webpage, allowing occupants to control it remotely. The lightweight IP (lwIP) (https://github.com/espressif/esp-lwip (accessed on 6 October 2024)) library for the ESP32 provides restricted access to network features. We developed a unique dataset named as the IDSH dataset [23] for intrusion detection in smart thermostats by using a Raspberry Pi-based adversary node to generate DoS and MITM attacks. The dataset is based on the features extractable from the smart thermostat using the lwIP library. This dataset is unique because existing datasets rely on packet capture tools (like Wireshark, tcpdump etc.) running on single-board computer-based gateways or workstations. In this work, we enhanced the performance of the IDS in terms of accuracy and computational efficiency through the novel implementation of CatBoost-based IDS on the ESP32 microcontroller. CatBoost implementation is not supported in existing ML libraries that port trained ML models to microcontrollers, so we quantized the trained CatBoost model to implement it in resource-constrained microcontrollers.

The main contributions of this work are as follows:

- A novel, computationally efficient, decentralized multi-class IDS using the CatBoost algorithm is proposed for deployment directly on IoT devices, without relying on gateways (such as Raspberry Pi or computers) or cloud computing. The decentralized multi-class IDS significantly improves detection accuracy by effectively handling categorical data and missing values. The system demonstrates reduced inference time on resource-constrained microcontrollers due to the fast prediction capability of the algorithm, ensuring that potential attacks are detected and mitigated with minimal latency. This enhances the robustness of the IDS across diverse IoT environments.
- Quantization of CatBoost model to fit models into the limited memory and processing capabilities of devices like the ESP32.
- A detailed comparison was conducted between XGBoost- and CatBoost-based IDS implementations on microcontroller-based IoT devices for binary and multi-class classification of DoS and MITM attacks using the IDSH dataset. The evaluation focused on both algorithms’ accuracy, inference time, and storage requirements.
- Additionally, we evaluated the impact of feature selection on the accuracy of the IDS and the computational burden on resource-constrained IoT devices.

The rest of the paper is organized as follows: Related work is presented in Section 2 and the proposed methodology is described in Section 3. The results are discussed in Section 4, followed by conclusion and future work in Section 5.

## 2. Related Work

The authors in [24] developed the permission and credibility-based IDS (PCIDS) using a decision tree (DT) for IoT devices to protect them against DoS and MITM attacks from adversaries. The PCIDS categorized the user requests into normal, abnormal, and risky categories by calculating the importance ratio, minimum credibility index, and maximum credibility index. The experimental results showed that the accuracy of PCIDS was 94.7%. The authors proposed to deploy the PCIDS on the gateway; however, hardware implementation of PCIDS on the gateway was not achieved.

In [25], the authors prevented the MITM attack on the Internet of Medical Things (IoMT) by transmitting the data signatures derived from locality-sensitive hashing (LSH). Furthermore, the authors also used the Hash Message Authentication Code (HMAC) to prevent modification attacks. The results showed that the proposed technique achieves a high detection rate with a false alarm rate (FAR) of 3%. In a recent study by Tekin et al. [26], the authors conducted a comparative analysis of the energy consumption of ML-based IDS on IoT devices, showing that on-device DT algorithms perform best in terms of training time, inference time, and power efficiency. However, the proposed technique was not tested in a real IoT environment.

In [27], a lightweight ML-based IDS is presented to be deployed on the edge side to detect impersonation attacks. Support vector machines (SVMs) were trained on the WiFi Intrusion Dataset to detect intrusions on the edge. The overall accuracy presented by this system was 98.22%. The authors in [18] introduced Deep-IDS for detecting DoS, DDoS, MITM, brute force, and replay attacks on Raspberry Pi-based edge servers/gateways. The Deep-IDS was trained using the CIC-IDS2017 dataset and achieved an accuracy of 97.67%. Similarly, a two-layered IDS was proposed in [28] to deploy IDS on Raspberry Pi-based the edge side and on cloud servers. In the first stage, an extra tree (ET)-based classifier was used for binary classification, and in the second stage, an ensemble technique was utilized which combined ET, RF, and deep neural network (DNN) for multi-classification. The proposed technique was tested on multiple datasets.

The IDS for real-time detection of DDoS attacks in smart homes based on software-defined networks (SDNs) was proposed in [29]. The authors collected the dataset of normal traffic and DDoS attacks, which was used to train SVM, Logistic Regression (LR), and DT. The results showed that DT outperformed other techniques with an accuracy of 99%.

The authors in [30] improved the precision of IDS using the Shapley (SHAP) value-based FS technique. The accuracy of the convolution neural network (CNN) and random forest (RF) using the FS technique for the CICIDS 2017 and NSL-KDD datasets was 98% with 10 features. In [31], the authors proposed a self-evolving host-based intrusion detection system (SEHIDS) that allows IoT devices to build their customized ANN model based on the traffic characteristics. The proposed technique used a multi-layer perceptron model for signature-based classification and a replicator neural network (ReNN) for anomaly-based intrusion detection. The authors tested their technique on the BoT-IoT, TON-IOT, and IoTID20 datasets. The authors claimed to have achieved an average true positive rate of 1. Similarly, the authors in [12] proposed a transformer-based network intrusion detection system (NIDS) by combining network traffic-based and data-based NIDS. The authors used the TON_IoT dataset and results showed that the proposed technique achieved 98.39% accuracy for binary classification and 97.06% for multi-classification. The authors in [32] proposed federated learning (FL)-based gated recurrent units (GRUs) NIDS for industrial IoT (IIoT). The accuracy of the training was improved by incorporating deep reinforcement learning (DRL). The results showed that the proposed technique performed better than non-FL and other variants of FL for intrusion detection.

In [33], the authors developed a lightweight IDS for IoT using cloud–edge collaboration. A stack-sparsed autoencoder (SSAE) was used for dimension reduction and a temporal convolution network (TCN) was utilized for intrusion detection. The proposed technique reduced the storage requirements, training time, and memory by 50% but the accuracy of the technique was approximately equal to centralized trained models.

Tiny ML is a subset of edge ML that allows the trained model to run on microcontrollers. In [34], the authors proposed a TinyML-based ECG classification system. They embedded a CNN model in the ECG device for classification, which reduces network bandwidth utilization and latency. The results showed that the accuracy of the TinyML-based classifier was 97%. The author in [35] embedded a unidirectional LSTM in a microcontroller for solar power forecasting and achieved a test R^2^ of 0.9590. In [36], the authors used TinyML for on-device anomaly detection by using the extreme value theory. The proposed technique achieved an excellent accuracy of 99.80%.

The literature reviewed above has explored the approaches of implementing IDS on gateways or on cloud servers. For successful IDS deployment on the edge, it must be both accurate and computationally efficient to detect attacks promptly. Most of the work has focused on deploying IDS on the edge (Raspberry Pi-based gateways) or in the cloud. However, IoT devices in smart homes are vulnerable to external threats due to their limited computational power and the absence of gateways. Therefore, there is a pressing need to develop IDS that can be embedded in IoT devices without relying on gateways or the cloud.

## 3. Materials and Methods

**Figure 1:**
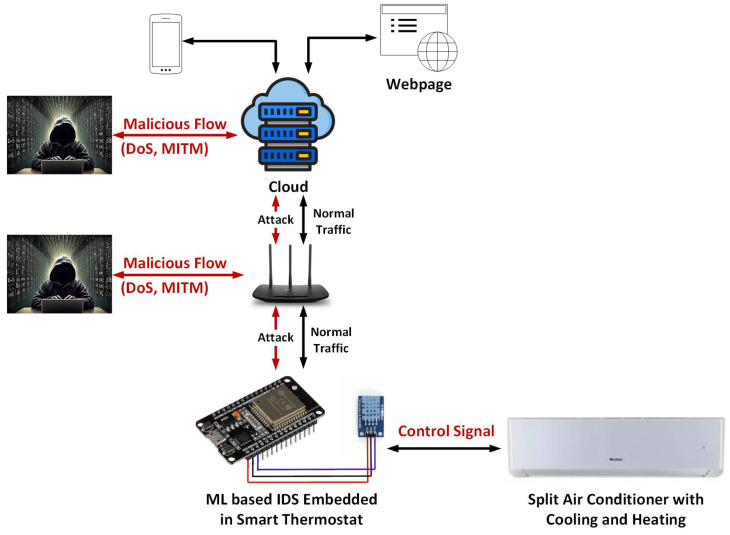
Proposed architecture of embedded IDS for smart thermostats.

In this work, we considered the application of smart thermostats in smart homes. The thermostat is designed to update the indoor air environment (temperature and humidity levels), and air conditioner control signal (ON/OFF) on an Amazon Web Service (AWS) server. The webpage hosted on the AWS server shows the indoor air environment parameters, and based on temperature and humidity levels, occupants can send control signals to the air conditioner through a webpage. We proposed to embed ML-based IDS in smart thermostats for on-device intrusion detection. The proposed architecture of embedded IDS is shown in Figure 1. The bi-directional communication between cloud and smart thermostats can be disrupted/manipulated by an adversary node. Since for most IoT devices, Raspberry Pi-based gateways are not available for the implementation of IDS, we proposed to embed an IDS in a smart thermostat in this work.

### 3.1. Threat Model

The adversary does not know the ML-based IDS is running on the smart thermostat. The adversary aims to intercept and modify the communication between the cloud and the smart thermostat. Moreover, the adversary can launch a DoS attack on the smart thermostat or cloud to make them irresponsive.

#### 3.1.1. Targeted System

The targeted system is a smart thermostat controlling the split air conditioner for maintaining indoor thermal comfort. The smart thermostat uploads the indoor air temperature to a website using WiFi and can be remotely controlled through HTTP.

#### 3.1.2. Adversarial Capabilities

The adversary node is implemented using Raspberry Pi 4 (manufactured by the Raspberry Pi Foundation, Cambridge, United Kingdom) with Kali Linux OS (version: 2023.3). The adversary can connect to the hotspot from which the smart thermostat is also connected and can generate DoS and MITM attacks as shown in Figure 1.

#### 3.1.3. Adversary Goals

The adversary’s primary objective is to compromise the communication between the smart thermostat and the cloud. The specific goals of the adversary include the following:

- Launching DoS attacks on either the smart thermostat or the cloud server to render them unresponsive.
- Eavesdropping on the communication between the smart thermostat and the cloud via MITM attacks.
- Manipulating the control signals sent to the air conditioner.
- Altering the temperature and humidity data uploaded to the webpage by the smart thermostat.

### 3.2. Training Dataset for IDS

Most of the well-known datasets like TON_IoT [37] and EdgeIIoT [38] used packet capture tools for dataset collection. These datasets required gateways to be part of the IoT architecture. The IDS proposed using such datasets is intended to be implemented on the gateway (Raspberry Pi). However, most IoT devices are connected to WiFi access points, and single-board computer (Raspberry Pi) gateways are not always available. We developed an IDSH dataset [23] that was captured on the ESP32 without using packet capture tools like Wireshark or tcpdump. We used the IDSH dataset to implement IDS on a smart thermostat for detecting DoS and MITM attacks. The dataset consists of sensor data, HVAC control signals, and network traffic features (connection activity and statistical data).

**Figure 2:**
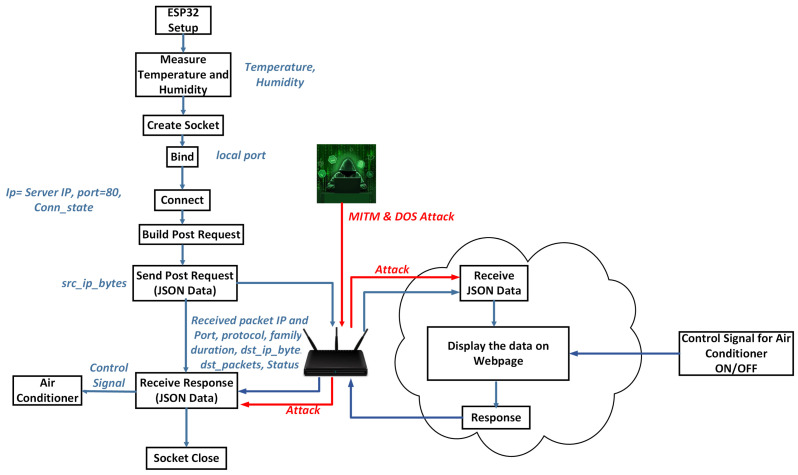
Dataset collection on smart thermostats.

The block diagram of the testbed is shown in Figure 2 and the parameters collected at each step are detailed in Figure 2. The steps involved in dataset collection are described in our previous work [19]. The IDSH dataset features description is given in Appendix A in Table A1. The IDSH dataset contains 4144 samples, which include instances of normal traffic, DoS attacks, and MITM attacks. The input features and target of IDS are shown in Table 1. The timestamp, source IP, and destination IP were excluded to prevent overfitting.

#### 3.2.1. Data Preprocessing

The dataset is normalized using the standard Min-Max normalization function, which scales the data to a range of [0, 1] for positive values or [−1, 1] when negative values are present. Label encoding is applied to convert the string-based output variables into integer format, enabling their use in training machine learning algorithms.

#### 3.2.2. Feature Selection

The chi-square [39] is used for FS to reduce the number of features. Table 2 shows the features selected by chi-square.

### 3.3. Deployment of a Machine Learning-Based Intrusion Detection System on a Smart Thermostat

In this study, XGBoost and CatBoost models are deployed on an ESP32-based smart thermostat for both binary and multi-class attack classification. The XGBoost-based IDS is implemented on the microcontroller using MicroMLgen [40]. Since existing libraries do not support the conversion of CatBoost models into optimized C++ code for microcontroller implementation, we quantized the CatBoost models to fit within the limited memory and processing capabilities of devices like the ESP32.

## 4. Results

In the absence of Raspberry Pi-based gateways, IoT devices have no defense against cyberattacks. Therefore, in this work, we embedded an ML-based IDS into a smart thermostat built with an ESP32 microcontroller. The ESP32 microcontroller has 440 KB of ROM and 520 KB of SRAM for program storage and execution. To implement the IDS, the smart thermostat extracts features from data traffic using the lwIP library. The smart thermostat communicates with an HTTP client hosted in the cloud through HTTP POST requests.

In our previous work [19], we evaluated the feasibility of embedding RF, XGBoost, DT, and ANN-based IDSs in terms of memory, inference time, and accuracy. Our results showed that XGBoost-based IDS outperformed DT, ANN, and RF for binary classification. In this work, we evaluated the performance of optimized CatBoost-based IDS on a smart thermostat for binary and multi-class classification and compared the results with our previous work. In this section, the implementation of IDS with CatBoost is discussed. Initially, we present the simulation results of CatBoost for binary and multi-classification of attacks on a smart thermostat. We also discuss the impact of FS on the performance of CatBoost implementation with reduced features using the FS technique. Later, we will discuss the implementation of CatBoost IDS on an ESP32-based smart thermostat and compare it with XGBoost.

### 4.1. CatBoost-Based IDS Implementation on IoT Device Without Feature Selection for Binary Classification

The performance of CatBoost for the implementation of IDS was evaluated for binary classification without using an FS technique. The IDSH dataset was split into 70% training and 30% testing. During training, 10-fold cross-validation was used to avoid overfitting and ensure generalization. The IDSH dataset has 20 input features while Label and Type are ground truths. The timestamp, source_ip, and destination_ip were not included as inputs to avoid overfitting. Hyperparameter tuning was performed using grid search. The parameters for grid search included depth, learning rate, regularization parameter (L2_Leaf_reg), and the number of trees. The range for the number of trees was [50, 100, 150, 200, 250, 300]; for 12_Leaf_reg, it was [1, 3, 5]; and the learning rate was [0.01, 0.1, 0.2, 0.3]. The performance of CatBoost was evaluated in terms of accuracy, precision, recall, and F1-score. The testing results are shown in Table 3. The highest accuracy of 99.03% was achieved at a depth of 10 with a false negative rate (FNR) of 1.30% and false positive rate (FPR) of 0.25%.

### 4.2. CatBoost-Based IDS Implementation on IoT Device with Feature Selection for Binary Classification

The chi-square [39] FS technique was used to find the optimal number of features. The FS technique was performed for each depth. We evaluated the performance of CatBoost for seven to sixteen features. Table 4 shows the best FS results for each depth. The results show that the CatBoost model with 10 features and a depth of 10 gives the highest accuracy of 99.03%.

### 4.3. Catboost-Based IDS for Multi-Classification Without Feature Selection

Similar to binary classification, for multi-classification, the timestamp, source_ip, and destination_ip were not included as inputs to avoid overfitting. Hyperparameter tuning was performed using grid search. The parameters for grid search included depth, learning rate, regularization parameter (L2_Leaf_reg), and the number of trees. The range for the number of trees was [50, 100, 150, 200, 250, 300]; for 12_Leaf_reg, it was [1, 3, 5]; and the learning rate was [0.01, 0.1, 0.2, 0.3]. The performance of CatBoost was evaluated in terms of accuracy, precision, recall, and F1-score. The testing results are shown in Table 5. The highest accuracy of 98.15% was achieved at a depth of seven.

### 4.4. Catboost-Based IDS for Multi-Classification with Feature Selection

The CatBoost model was also trained for multi-classification by using the FS technique. We evaluated the performance of CatBoost with seven to sixteen features. Below seven features, the accuracy of IDS dropped below 90%. The performance of CatBoost was evaluated for depths from six to ten and the best performance at each depth with FS is shown in Table 6. The results show that CatBoost with depth=7 and with 10 features gave highest accuracy of 98.15%.

### 4.5. Quantization of CatBoost-Based IDS

Quantization in ML is used for compressing large model data. The ESP32 has a limited memory and processing power; therefore, running a large ML model may not be feasible or may require a large inference time. The weights and parameters of trained CatBoost models are saved in double format for higher precision. The floating point numbers can be represented in half (16-bit), single (32-bit), and double (64-bit) precision. The hardware acceleration in ESP32 is only supported for single precision, while double is implemented using software that requires more memory and takes longer to complete the operation. Therefore, in this work, we utilized post-training quantization in which the model parameters and weights of the CatBoost model were stored using single precision. Quantization may result in a small decrease in model accuracy due to reduced numerical precision. However, in CatBoost models, where tree-based learning creates discrete splits, the impact is generally minimized. This trade-off is often acceptable for models that do not rely heavily on high precision in calculations, particularly for inference tasks on ESP32. Since the ESP32 supports hardware acceleration for single precision (32-bit float), converting the parameters of CatBoost from double to float results in lower memory consumption and faster inference time.

**Figure 3:**
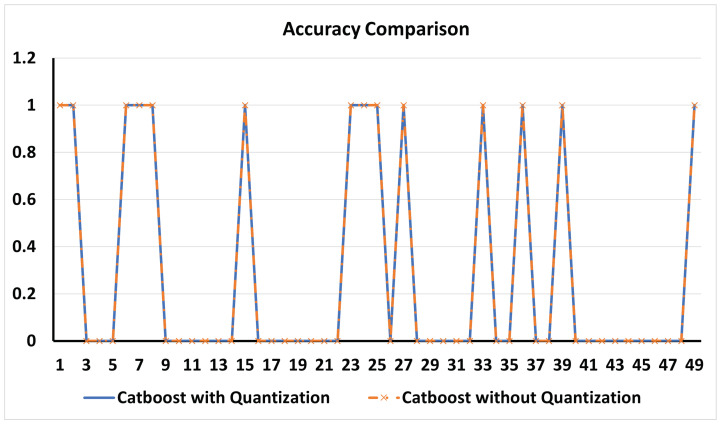
Comparison of IDS implemented with quantization and without quantization.

Therefore, to support our aforementioned discussion, we also compared the detection accuracy of CatBoost (Depth = 6, Trees = 200) with and without quantization for binary classification in Figure 3 and the results illustrate that the accuracy did not decrease. In Figure 3, the graph shows the detection of benign (0) and attack (1) for 50 samples. The CatBoost with quantization results are the results of the model running on ESP32 and the CatBoost without quantization results are the results of the model running on the local machine. Therefore, we can conclude that applying quantization for limited memory-based ESP32 did not compromise the accuracy of the detection.

### 4.6. IDS Implementation on Smart Thermostat for Real-Time Intrusion Detection

The implementation of IDS on a smart thermostat is challenging due to its limited memory and processing power. In our previous work, we implemented an XGBoost-based IDS for binary classification without using an FS technique. In this section, we evaluate the implementation of a CatBoost-based IDS on a smart thermostat for both binary and multi-class classification. Additionally, we compare the performance of the CatBoost-based IDS with the XGBoost-based IDS on the smart thermostat.

The ESP32 has limited RAM and program storage, which prevents embedding an IDS with a large number of trees and deep models. In Table 7, for Depth = 6, a CatBoost model with 200 trees can be embedded for binary classification. Beyond 200 trees, the memory overflows on the ESP32. For Depth = 7, a CatBoost model with 120 trees can be embedded without using FS, and with FS, a model with 140 trees can be embedded. Similarly, the implementation results for the maximum possible number of trees and depths are shown in Table 7. The results show that for binary classification, the CatBoost-based IDS achieved the highest accuracy of 98.71%, outperforming the XGBoost-based IDS developed in our previous work [19], which had an accuracy of 97.66%. The results indicate that the CatBoost-based IDS is more accurate and faster than the XGBoost-based IDS.

For multi-class classification, the highest accuracy of 97.51% was achieved by the CatBoost-based IDS (90 trees, depth = 6). In comparison, the highest accuracy achieved by the XGBoost-based IDS (50 trees, depth = 7) was 96.70%, with an inference time of 2111 μs, whereas the inference time for CatBoost was only 267 μs.

### 4.7. Discussion

In this study, we developed an IDS for IoT devices in smart homes where gateways are not available for the deployment of IDS. The IDS was embedded in a smart thermostat for real-time intrusion detection without relying on gateways.

**Figure 4:**
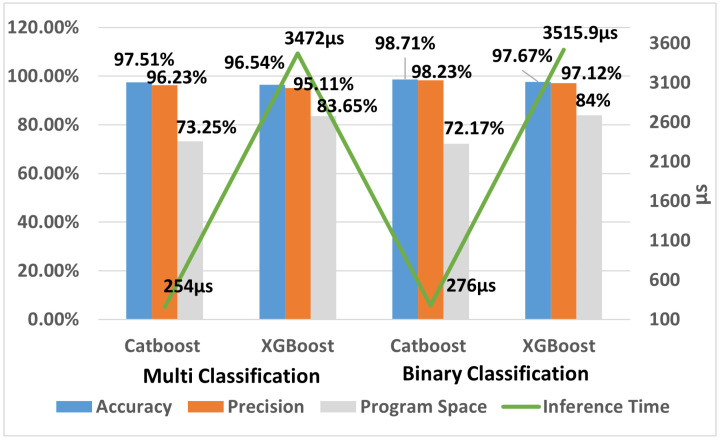
Comparison of IDS implemented with CatBoost and XGBoost on the smart thermostat.

We evaluated the feasibility of implementing the IDS for binary and multi-class classification. The CatBoost model (Trees = 200, Depth = 10) achieved the highest accuracy of 99.03% for binary classification both with and without FS. However, due to the limited program storage and RAM available on the ESP32, embedding a CatBoost model with a depth greater than eight was not possible. The IDS performance comparison of XGBoost and CatBoost for binary and multi-classification on smart thermostat is shown in Figure 4. The highest number of trees allowed for Depth = 7 was 140, and for Depth = 8, the maximum number of trees was 70. Therefore, the maximum accuracy achieved by the CatBoost-based IDS on the smart thermostat was 98.71% using a depth of six and 200 trees. The CatBoost-based IDS outperformed the IDS implemented with XGBoost in our previous work [19] in terms of accuracy, inference time, and program storage. The CatBoost-based IDS improved accuracy by 1.06%, decreased inference time by 92.14%, and reduced program storage by 14.09%.

Similarly, for multi-class classification, the highest accuracy was 98.15% with the CatBoost (Depth = 7, Trees = 200)-based IDS. On the smart thermostat, the highest accuracy of the CatBoost-based IDS was 97.51%. The CatBoost model outperformed the XGBoost-based IDS in terms of accuracy, inference time, and program storage. The CatBoost-based IDS improved accuracy by 0.83%, reduced inference time by 87.35%, and reduced program storage by 11.32%.

The increase in the number of trees improves accuracy by capturing complex patterns, but this increase also raises the computational burden; as a result, inference time and memory consumption increase. A deeper CatBoost/XGBoost model captures complex patterns and increases accuracy. However, deeper trees consume more memory and increase inference time. As shown in Table 3, increasing tree depth generally improves accuracy. However, in memory-limited devices such as ESP32 microcontrollers, Table 7 demonstrates that as depth increases, fewer trees can be used before memory overflow occurs. For example, at a depth of six, the maximum number of trees is 200 for binary classification and 90 for multi-classification. When depth is increased to eight, the maximum number of trees drops to 70 for binary classification and 25 for multi-classification.

The FS method improved the accuracy for binary classification by using a reduced number of features. Table 4 shows that with a reduced number of features, the accuracy improves. However, on the microcontroller, the FS technique did not reduce inference time or program storage. The inference time depends on the number of trees and the depth of the trees. Since the number of trees was either the same or greater, the inference time was not significantly improved.

CatBoost and XGBoost are both based on the gradient boosting framework, using decision trees as base learners. However, CatBoost is particularly well-suited for constrained devices like the ESP32 due to its native support for categorical data, eliminating the need for preprocessing. CatBoost’s ordered boosting technique efficiently handles training and inference without storing large amounts of data, making it ideal for memory-limited environments.

Additionally, CatBoost prevents target leakage by splitting the data into multiple parts and using only past data for predictions. This approach not only maintains high accuracy but also reduces storage requirements and prevents overfitting as depicted in Figure 4. Unlike XGBoost, CatBoost uses symmetric trees, which require fewer computations for predictions, contributing to faster inference times. This is critical for implementing IDS on resource-constrained devices like the ESP32, where minimal inference time is crucial.

Furthermore, CatBoost’s symmetric tree structure allows it to use fewer trees and shallower depths while maintaining performance, which further enhances inference speed as shown in Table 7. These characteristics make CatBoost a more efficient choice than XGBoost for IDS deployment on microcontrollers.

In smart homes, IoT devices have limited computational resources and are used in sensitive applications like door locks, CCTVs, and smart thermostat, etc. During the interaction with the cloud, an adversary can stop the operation of these devices with a DoS attack or can intercept the sensitive information through an MITM attack. A lightweight IDS that can quickly and efficiently detect these attacks with minimal computational load can enhance security for homeowners. Rapid detection of DoS attacks is especially crucial, as it allows for mitigation before the device is overwhelmed. In this work, we demonstrate that a CatBoost-based IDS can detect these attacks in under 276 microseconds, without sacrificing accuracy.

## 5. Conclusions and Future Work

In this study, we proposed a CatBoost-based IDS for IoT devices in smart homes. The system is designed to work with IoT devices that communicate with the cloud, eliminating the need for resource-intensive gateways like Raspberry Pi or computers. Our embedded machine learning-based IDS effectively protects these devices from DoS and MITM attacks without relying on gateways or cloud processing. The results showed that the CatBoost-based IDS achieved 99.03% accuracy for binary classification and 98.15% accuracy for multi-class classification. Due to the limited processing power and memory of the ESP32-based smart thermostat, the highest achieved accuracy was 98.71% for binary classification and 97.51% for multi-class classification. The CatBoost IDS performed binary classification in 276 μs and multi-class classification in 273 μs. Additionally, CatBoost-based IDS outperformed the XGBoost-based IDS in terms of accuracy, program storage, and inference time. While FS methods slightly improved accuracy, the inference time remained unaffected, as the number of trees and tree depth did not change.

For future work, we plan to expand the IDSH dataset to include more attack types and a larger sample size. Due to the limited resources and restricted access to network parameters in the lwIP library, we were able to extract only a limited set of network features from the microcontroller. Moving forward, we aim to extract a wider range of network parameters directly from IoT devices to further improve the IDS’s effectiveness. Additionally, we will explore other machine learning techniques that require fewer computational resources while delivering higher accuracy.

## Figures and Tables

**Table 1:** Inputs and target of IDS.

Selected Input Features	Target
source_port, dst_port, protocol, Family, Service, Duration, source_bytes, destination_bytes, conn_state, missed_bytes, source_packets, src_ip_bytes, destination_packets, dst_ip_bytes, Temperature, Humidity, Status	Label (binary classification), Type (multi-class classification)

**Table 2:** Selected features.

No. of Features	Selected Features
7	source_port, Duration, missed_bytes, dst_ip_bytes, Temperature, Humidity, Status
8	source_port, Duration, destination_bytes, missed_bytes, dst_ip_bytes, Temperature, Humidity, Status
9	source_port, Duration, destination_bytes, conn_state, missed_bytes, dst_ip_bytes, Temperature, Humidity, Status
10	source_port, protocol, Duration, destination_bytes, conn_state, missed_bytes, dst_ip_bytes, Temperature, Humidity, Status
11	source_port, protocol, Duration, source_bytes, destination_bytes, conn_state, missed_bytes, dst_ip_bytes, Temperature, Humidity, Status

**Table 3:** Performance of CatBoost for binary classification.

Hyperparameters	Accuracy	Precision	Recall	F1-Score	FPR	FNR
Trees = 200, Depth = 6, 12_Reg_Leaf = 1, LR = 0.1	98.63%	0.98	0.98	0.98	1.41%	1.25%
Trees = 200, Depth = 7, 12_Reg_Leaf = 3, LR = 0.3	98.79%	0.98	0.99	0.99	1.30%	1.00%
Trees = 200, Depth = 8, 12_Reg_Leaf = 3, LR = 0.3	98.79%	0.98	0.98	0.98	1.41%	0.75%
Trees = 200, Depth = 9, 12_Reg_Leaf = 3, LR = 0.3	98.63%	0.98	0.98	0.98	1.30%	1.50%
Trees = 200, Depth = 10, 12_Reg_Leaf = 3, LR = 0.3	99.03%	0.99	0.99	0.99	1.30%	0.25%

**Table 4:** Performance of CatBoost for binary classification using feature selection.

Hyperparameters	No. of Features	Accuracy	Precision	Recall	F1-Score	FPR	FNR
Trees = 200, Depth = 6, 12_Reg_Leaf = 3, LR = 0.3	9	98.71%	0.98	0.99	0.98	1.53%	0.75%
Trees = 200, Depth = 7, 12_Reg_Leaf = 3, LR = 0.3	9	98.87%	0.98	0.98	0.98	1.41%	1.00%
Trees = 200, Depth = 8, 12_Reg_Leaf = 3, LR = 0.3	9	98.95%	0.98	0.99	0.99	1.18%	0.75%
Trees = 150, Depth = 9, 12_Reg_Leaf = 3, LR = 0.3	9	98.87%	0.98	0.99	0.99	1.18%	1.00%
Trees = 200, Depth = 10, 12_Reg_Leaf = 3, LR = 0.3	10	99.03%	0.99	0.99	0.99	1.30%	0.25%

**Table 5:** Performance of CatBoost for multi-classification without using feature selection.

Hyperparameters	Accuracy	Precision	Recall	F1-Score
Trees = 250, Depth = 6, 12_Reg = 3, LR = 0.3	97.90%	0.96	0.96	0.96
Trees = 200, Depth = 7, 12_Reg_Leaf = 3, LR = 0.3	98.15%	0.97	0.97	0.97
Trees = 250, Depth = 8, 12_Reg_Leaf = 3, LR = 0.3	97.90%	0.97	0.96	0.96
Trees = 150, Depth = 9, 12_Reg_Leaf = 3, LR = 0.3	97.90%	0.96	0.96	0.96
Trees = 250, Depth = 10, 12_Reg_Leaf = 3, LR = 0.3	97.66%	0.96	0.96	0.96

**Table 6:** Performance of CatBoost for multi-classification with feature selection.

Hyperparameters	No. of Features	Accuracy	Precision	Recall	F1-Score
Trees = 250, Depth = 6, 12_Reg_Leaf = 3, LR = 0.3	10	97.91%	0.97	0.96	0.96
Trees = 200, Depth = 7, 12_Reg_Leaf = 3, LR = 0.3	10	98.15%	0.97	0.97	0.97
Trees = 250, Depth = 8, 12_Reg_Leaf = 3, LR = 0.3	10	97.91%	0.97	0.96	0.96
Trees = 150, Depth = 9, 12_Reg_Leaf = 3, LR = 0.3	10	97.91%	0.96	0.96	0.96
Trees = 150, Depth = 10, 12_Reg_Leaf = 3, LR = 0.3	8	97.74%	0.96	0.96	0.96

**Table 7:** Implementation of IDS on the smart thermostat for binary and multi-classification.

ML Models (Hyperparameters)	No. of Features	Classification	Accuracy	Inference Time μs (Average)	Program Storage
CatBoost (Trees = 200, Depth = 6, 12_Reg_Leaf = 3, LR = 0.3)	17	Binary	98.63%	284	72.20%
CatBoost (Trees = 120, Depth = 7, 12_Reg_Leaf = 3, LR = 0.3)	17	Binary	98.55%	223	72.83%
CatBoost (Trees = 70, Depth = 8, 12_Reg_Leaf = 3, LR = 0.3)	17	Binary	98.47%	249	71.85%
CatBoost (Trees = 200, Depth = 6, 12_Reg_Leaf = 3, LR = 0.3)	9	Binary	98.71%	276	72.17%
CatBoost (Trees = 140, Depth = 7, 12_Reg_Leaf = 1, LR = 0.3)	9	Binary	98.47%	260	73.59%
CatBoost (Trees = 70, Depth = 8, 12_Reg_Leaf=1, LR = 0.3)	10	Binary	98.47%	253	71.76%
XGBoost (Trees = 100, Depth = 8) [19]	17	Binary	97.66%	3515	84.01%
CatBoost (Trees = 90, Depth = 6, 12_Reg_Leaf = 3, LR = 0.3)	17	Multi-Classification	97.51%	267	73.25%
CatBoost (Trees = 90, Depth = 6, 12_Reg_Leaf = 3, LR = 0.3)	10	Multi-Classification	97.51%	273	73.25%
CatBoost (Trees = 40, Depth = 7, 12_Reg_Leaf = 3, LR = 0.3)	17	Multi-Classification	96.86%	160	72.86%
CatBoost (Trees = 40, Depth = 7, 12_Reg_Leaf=3, LR = 0.3)	10	Multi-Classification	96.86%	158	72.51%
CatBoost (Trees = 25, Depth = 8, 12_Reg_Leaf = 3, LR = 0.3)	17	Multi-Classification	96.86%	140	71.38%
CatBoost (Trees = 25, Depth = 8, 12_Reg_Leaf = 3, LR = 0.3)	10	Multi-Classification	96.86%	137	71.27%
XGBoost (Trees = 100, Depth = 6, LR = 0.3)	17	Multi-Classification	96.14%	4075	85.68%
XGBoost (Trees = 100, Depth = 6, LR = 0.3)	10	Multi-Classification	96.14%	4073	85.68%
XGBoost (Trees = 50, Depth = 7, LR = 0.3)	17	Multi-Classification	96.30%	2011	80.38%
XGBoost (Trees = 50, Depth = 7, LR = 0.3)	7	Multi-Classification	96.54%	2037	81.12%
XGBoost (Trees = 50, Depth = 8, LR = 0.3)	17	Multi-Classification	96.54%	3472	83.65%
XGBoost (Trees = 50, Depth = 7, LR = 0.3)	7	Multi-Classification	96.70%	2111	82.60%

## Data Availability

The dataset presented in the study is openly available on Kaggle at https://doi.org/10.1109/JIOT.2023.3289206 (accessed on 8 October 2024).

## Appendix A

**Table A1:** Description of the IDSH dataset.

ID	Feature	Type	Description
1	timestamp	Time	Timestamp of connection
2	source_ip	String	Source IP address
3	source_port	Number	Source ports which originate endpoint’s TCP/UDP ports
4	destination_ip	String	Destination IP address
5	dst_port	Number	Destination ports which respond to endpoint’s TCP/UDP ports
6	protocol	String	Transport layer protocols of flow connections
7	Family	String	Address family
8	Service	String	Dynamically detected protocols, such as DNS, HTTP, and SSL
9	Duration	Time	The difference in the time between the packet being sent from the source and receiving the response from the cloud server
10	source_bytes	Number	Source bytes which originate from the source
11	destination_bytes	Number	Destination bytes which are responses from the destination
12	conn_state	String	Various connection states, such as S0 (connection without replay), S1 (connection established), and REJ (connection attempt rejected)
13	missed_bytes	Number	Number of missing bytes in content gaps
14	source_packets	Number	Number of original packets which is estimated from source systems
15	src_ip_bytes	Number	Number of original IP bytes which is the total length of IP header field of source systems
16	destination_packets	Number	Number of destination packets which is estimated from destination systems
17	dst_ip_bytes	Number	Number of destination IP bytes which is the total length of IP header field of destination systems
18	Temperature	String	Room temperature (°C) of the Zone
19	Humidity	String	Humidity (%) of the Zone.
20	Status	String	Status of air conditioner (ON/OFF).
21	Label	Number	Tag Normal traffic as 0 and attack as 1.
22	Type	String	Tag attack categories as Normal, DoS, and MITM.
